# Elevation of corticosterone and 17OH progesterone in extremely preterm infants and clinical implications

**DOI:** 10.1038/s41390-025-04216-5

**Published:** 2025-08-07

**Authors:** Julie M. Kessel, Patrice K. Held, Eric R. Bialk, Ryan M. McAdams, Ian M. Bird

**Affiliations:** 1 Dept of Pediatrics, University of Wisconsin School of Medicine and Public Health, Madison, WI USA; 2 Department of Molecular and Medical Genetics, Oregon Health and Science University, Portland, Oregon, OR USA; 3 Wisconsin State Laboratory of Hygiene and Department of Biostatistics and Medical Informatics, University of Wisconsin School of Medicine and Public Health, Madison, WI USA; 4 Department of Obstetrics and Gynecology, University of Wisconsin School of Medicine and Public Health, Madison, WI USA

## Abstract

**Background:**

A greater understanding of gestational age-adjusted steroid profiles in preterm neonates is needed to inform diagnosis, optimal timing, dosage, and duration of postnatal steroid therapy for preterm infants with adrenal insufficiency. Therefore, we evaluated changes in steroid profiles using newborn screening dried blood spots (DBS) to determine the impact of prematurity on fetal adrenal function.

**Methods:**

Cortisol and its biosynthetic precursors were quantified in 813 anonymized DBS from newborns between 23 to 42 weeks of gestation, collected within 72 hours of birth and again approximately two weeks later. Steroid quantification was performed using liquid chromatography-tandem mass spectrometry (LC-MS/MS), with analyses stratified by gestational and postnatal ages.

**Results:**

Extremely preterm infants often had elevated cortisol levels, with a general decline as gestational age increased. Levels of 17-hydroxyprogesterone (17OHP4) and corticosterone were significantly higher in most preterm newborns, with 17OHP4 showing the strongest inverse correlation with gestational age. In contrast, aldosterone levels remained unaffected by gestational age.

**Conclusions:**

Elevations in 17OHP4 and corticosterone, rather than cortisol alone, reflect prematurity’s effect on adrenal sufficiency. Preterm infants with high cortisol and elevated corticosterone and 17OHP4 levels may struggle to meet their physiological cortisol needs through the hypothalamic-pituitary-adrenal axis, increasing the risk of adrenal crises. These findings provide important insights to guide the management of adrenal insufficiency in preterm neonates.

**Impact:**

The increasing survival of preterm infants born at earlier gestational ages presents clinicians with the complex challenge of distinguishing normal from abnormal adrenal function, a determination which may be critical for survival. Our study advances the understanding of adrenal dysfunction in this population by employing analytical methods rooted in the steroid biosynthesis pathway’s maturation. By utilizing techniques traditionally used to diagnose congenital adrenal hyperplasia (CAH), we provide actionable guidance for interpreting steroid data in preterm infants, offering a practical framework for improved clinical decision-making.

## Introduction

Medical advancements now enable the survival of preterm infants as early as 22 weeks gestational age (GA), necessitating a better understanding of their unique physiological challenges, including adrenal function, which is pivotal for survival. Research on adrenal function lags behind other systems, such as pulmonary development. Unlike pulmonary maturation induced by betamethasone, adrenal function is transiently suppressed by adrenocorticotropic hormone (ACTH) blockade,^1^ potentially leading to life-limiting consequences. Adrenal immaturity may be associated with early-onset adrenal insufficiency, including hypotension and respiratory distress^2–6^ Recent reports have also described late-onset adrenal insufficiency, marked by hypotension and electrolyte imbalance.^7,8^ Therefore, a deeper understanding of adrenal physiology, particularly cortisol and aldosterone synthesis, is essential for diagnosing and managing adrenal insufficiency in preterm neonates.

Cortisol levels are commonly used to assess adrenal function, but its utility in preterm neonates is limited due to variability and inconsistent correlations with clinical outcomes.^9–14^ Studies report similar basal cortisol levels in preterm and full-term infants, without reliable correlations to health status or adrenal insufficiency.^8,14–16^ Furthermore, studies of steroidogenic precursors face methodological challenges, like immunoassay cross-reactivity.^14^ Therefore, relying solely on cortisol may be inadequate, underscoring the need for comprehensive steroid profiling that includes steroidogenic precursors and end products, considering hypothalamic-pituitary-adrenal (HPA) axis development.

**Fig. 1:**
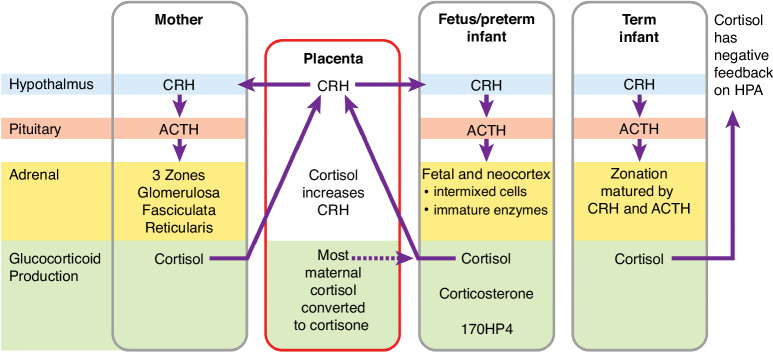
The immature hypothalamic-pituitary-adrenal axis of the fetus and preterm infant responds dynamically to birth and stress. Going from left to right on the panels, the hypothalamic (blue) and pituitary (pink) stimuli and adrenal structure are compared in the mother, fetus/preterm infant and term infant. The placenta is included to highlight this organ as an important source of CRH during gestation and as a site for conversion of most maternal cortisol to cortisone. Focusing on the adrenal gland (yellow), the maternal adrenal with 3 well defined zones is compared to the developing adrenal of the fetus, preterm and term infant. In the fetus and preterm infant, the fetal and neocortex adrenal cells are intermixed, and steroid biosynthetic enzyme function is immature.^18,21^ In the term infant, ACTH and CRH stimulation induces both zonation and maturation of enzymes allowing increased synthesis of cortisol. Glucocorticoid production (green) is compared to highlight that the major products of the disorganized and immature fetal and preterm adrenal include corticosterone and 17OPH4 in addition to cortisol.

Fetal adrenal development begins early, influenced primarily by corticotropic releasing hormone (CRH) and ACTH. Cortisol facilitates cross talk between mother, placenta, and fetus, signaling readiness for birth (Fig. 1).^17^ Initially, the fetal adrenal gland is disproportionately large with underdeveloped zonation.^18^ Unlike the distinct zones of an adult adrenal cortex, the fetal gland features a neocortex with mixed cell types and incomplete zonation.^18^ By 22-25 weeks GA, the fetal zone is more than twice the size of the adult zone. In the third trimester, ACTH and CRH surges induce zonation and enzyme functionality in the adrenal glands, enabling independent cortisol and aldosterone production post-birth.^19^ Consequently, infants born pre-third trimester exhibit notable differences in adrenal size, structure, and function compared to term infants.

**Fig. 2:**
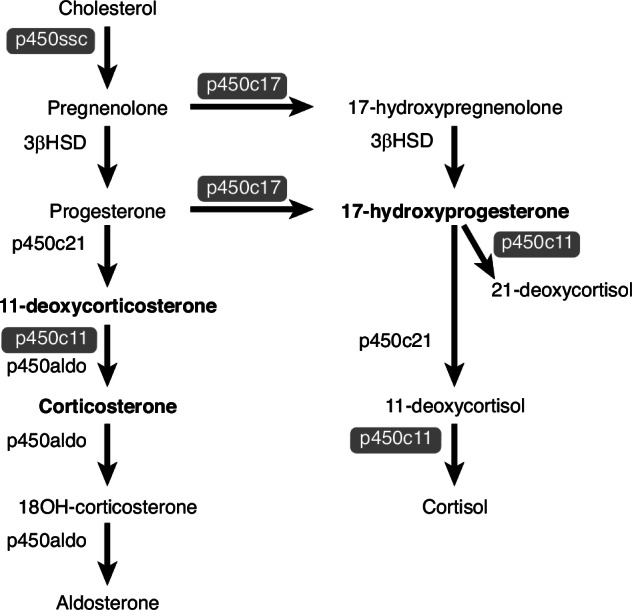
Mineralocorticoid and glucocorticoid synthesis by the fetal/preterm adrenal. Adrenal steroid precursors and biosynthetic enzymes are shown for the mineralocorticoid pathway that synthesizes aldosterone and the glucocorticoid pathway that synthesizes cortisol. In the fetus and preterm infant in the absence of ACTH simulation, enzymes highlighted in grey have not been fully induced by cAMP-coupled hormone mediators and as such, have limited affinity for substrate in the fetal and extremely preterm adrenal.^18,21^ Enzymes with limited affinity include p450ssc, p450c11, and p450c17. Consequently, and in parallel to congenital enzyme deficiencies, steroid intermediates in bold, including 17-hydroxyprogesterone (17OHP4), 11-deoxycorticosterone, and corticosterone are increased in the fetus and preterm infant compared to term infant and adult adrenal, which converts pregnenolone to cortisol with >99% efficiency. 17OHP4 is the steroid intermediate measured in DBS for congenital adrenal hyperplasia newborn screening. 17OHP4 is elevated in preterm infants due to P450c11 insufficiency. Corticosterone is elevated due to P450c17 insufficiency.

Adrenal enzyme function remains immature at 22-25 weeks GA, a fact often overlooked in developmental studies.^20^ As shown in Fig. 2, key enzymes for cortisol and mineralocorticoid synthesis include p450c17 (17 alpha hydroxylase), 3βHSD (3β hydroxysteroid dehydrogenase), p450c11 (11β hydroxylase), p450aldo (aldosterone synthase, also p450c18AS), and p450c21 (21-hydroxylase). Late in gestation, p450c17 remains a limiting factor in shifting production of corticosterone to cortisol.^21,22^ During the third trimester, ACTH surges enhance both adrenal zonation and enzyme functionality, particularly p450c17,^23,24^ thereby boosting cortisol production to prepare the fetus for birth and postnatal life. The absence of this ACTH surge before preterm delivery may result in cortisol deficiency and an accumulation of steroid precursors.

ACTH and CRH modulate adrenal enzyme expression essential for cortisol and aldosterone production (Fig. 1).^1,25^ In preterm newborns, stress-triggered ACTH release predominantly increases cortisol rather than aldosterone production, reflecting the limited adrenal capacity for aldosterone synthesis.^26^ Variations in fetal HPA axis, neocortex size, and adrenal enzyme expression influence steroid profiles between preterm and full-term infants. In preterm infants, immature adrenal development may restrict adequate cortisol production and feedback, increasing cortisol precursors. Under clinical stress, this imbalance could mimic congenital adrenal deficiencies, presenting with hypotension and electrolyte imbalances.

Steroidogenic precursors within the glucocorticoid pathway can be measured in dried blood spots (DBS) collected for universal newborn screening. Elevated 17OHP4, a pivotal intermediate (Fig. 2), is indicative of genetic enzyme deficiencies such as 21-hydroxylase deficiency (p450c21). Newborn screening programs test over 95% of infants within the first 48 hours of birth for congenital disorders requiring immediate intervention.^27,28^ Preterm infants, due to their vulnerability, often undergo multiple screenings.^24,29^ Elevated baseline 17OHP4 levels in preterm infants, even without genetic deficiencies, underscore the importance of establishing GA-specific thresholds.^13,30,31^ However, the diagnostic and management roles of steroid precursors such as 17OHP4 and corticosterone in preterm adrenal insufficiency remain underexplored.

Analyzing steroidogenic precursors like 17OHP4 and corticosterone provide insights into incomplete fetal adrenal development. Elevated 17OHP4 suggests strong ACTH drive with adrenal inefficiency at p450c21 or p450c11. Increased corticosterone indicates insufficient p450c17. A low cortisol/corticosterone ratio reflects insufficient p450c17, while a higher ratio signifies abundant enzyme expression and efficient cortisol production.

In this study, we analyzed the cortisol/corticosterone ratio and early pathway markers (17OHP4 and corticosterone) to track fetal adrenal development at two time points: within 72 hours after birth and again at 10-14 days after delivery. Our goal was to determine if extremely preterm infants’ reduced cortisol production efficiency underlies their critical condition and identify specific GAs most affected. Studying adrenal physiology in extremely preterm infants is challenging due to the significant blood volume required for cortisol and precursor analysis. However, LC-MS/MS assays in newborn screening labs offer a method for analyzing extensive steroid precursors from small DBS volumes, potentially improving screening for congenital adrenal hyperplasia.^24,30,32,33^ Using a large cohort of newborn screening specimens and advanced steroid analysis, this study documents steroid concentration changes due to adrenal insufficiency and extreme prematurity, providing key insights into adrenal development and the clinical utility of biomarkers for managing adrenal insufficiency in preterm newborns.

## Materials and Methods

### Specimens

Protocol (2019-0118) for the use of residual DBS newborn screen specimens was approved by the Health Sciences Institutional Review Board at the University of Wisconsin (March 28, 2019).

For the analysis, 813 de-identified residual DBS were used, with demographic data including sex, birthweight, GA, and sample collection timing. Most specimens (82.4%) were collected within 0-72 hours (early) after birth, with the rest (17.6%) collected between 10–18 days. The GAs spanned from 23 to 44 weeks, with half of infants born at ≤36 weeks. Early samples showed an even distribution across birth weight categories, while later samples had more infants ≤1500 gm, aligning with newborn screening protocols. The cohort’s balanced representation of various GAs and birth weights ensured a comprehensive study scope (Table 1).

**Table 1:** Newborn screening specimen demographic data distribution.

		Collection 0–72 hours			Collection 10–18 days
Count		670 (82%)			143 (18%)
Timing of Collection		0–23 h	24–48 h	49–72 h	
		71 (11%)	550 (82%)	49 (7%)	
Gestational Age (weeks)	23–25	65 (10%)			29 (20%)
	26–28	85 (13%)			63 (44%)
	29–32	78 (12%)			31 (22%)
	33–36	124 (19%)			19 (13%)
	37–42	318 (47%)			1 (1%)
Birth weight	<=1500 g	188 (28%)			117 (82%)
	1501–2499 g	244 (36%)			23 (16%)
	>=2500 g	238 (36%)			3 (2%)
Sex	M:F	333 (50%):337 (50%)			75 (52%):68 (48%)

Due to the retrospective analysis of a large cohort of newborns, access to patient charts was neither permissible nor feasible, and therefore, the study members were unable to assess the impact of pre- and postnatal steroid treatments on the concentration of cortisol and steroid intermediates.

### Quantification of cortisol and steroid intermediates

The steroid profiling assay, previously described by Held et al., was modified to include a total of seven steroids (cortisol, 17OHP4, androstenedione, 11-deoxycortisol, aldosterone, corticosterone, and progesterone). In summary, the 7 steroids were extracted from a 3.2 mm (1/8”) punch of the DBS specimen using a solvent of 80/20 acetonitrile/water containing internal standards (concentration of ~3 ng/mL for each steroid internal standard). The solution was then dried under nitrogen gas and reconstituted using a 40/60 methanol/water solution with 0.3% formic acid. The steroids were separated by liquid chromatography using a Phenomenex Kinetex (Torrance, CA) 5 μm C18 110 A 50 × 2.1 mm column and analyzed using an AB Sciex API 4500 tandem mass spectrometer with a TurboV electrospray ionization source in positive mode using multiple reaction monitoring. Supplemental Table 1 provides the MRM (multiple reaction monitoring) transitions, retention times, and collision energy for each steroid and its corresponding internal standard. Of note, postnatal treatment with hydrocortisone cannot be distinguished from endogenous cortisol production due to identical mass-to-charge ratios and retention times.

Adequate baseline separation of the 7 steroids was achieved with a run time of 13.5 minutes. Supplemental Table 2 provides the liquid chromatography injection profile for the separation of the steroids. Steroid concentrations were determined using the quantifying transitions and a linear regression curve with no weighting (forced through zero) and are reported in ng/mL of whole blood (DBS) throughout the manuscript. One unit of whole blood in a DBS is equal to 2 units of serum, thereby serum concentrations can be calculated by multiplying the DBS value by 2 and converting into appropriate units (ug/dL serum).

All data acquisition and processing were performed using Analyst 1.6.2 software (AB Sciex, Redwood City, CA).

### Statistical analysis of data

Statistical analyses stratified cortisol data by GA and newborn age at sampling. Mean, median, and standard deviation were calculated for each group. The Mann-Whitney test assessed differences, considering the non-parametric nature of the data. Cortisol and steroid intermediates, plotted against GA for the initial 72 hours post-birth, underwent Spearman’s correlation to examine their relationship with GA, where the Spearman’s coefficient ranges from -1 to 1, indicating perfect inverse or direct correlations, respectively. Negative coefficients suggest a decreasing trend with GA, and positive coefficients indicate an increasing trend.

## Results

The analysis explored the relationship between steroid analyte and GA over the study period, to allow a visual representation of the data spread with prematurity. Relationships were assessed using Spearman’s correlation coefficient, where significant positive or negative coefficient values suggested changes in steroid levels occurred with GA. The statistical significance of these correlations is shown in the accompanying figures. Cortisol data were further stratified by GA cohorts to provide comparisons with findings from other studies.

### Aldosterone

**Fig. 3:**
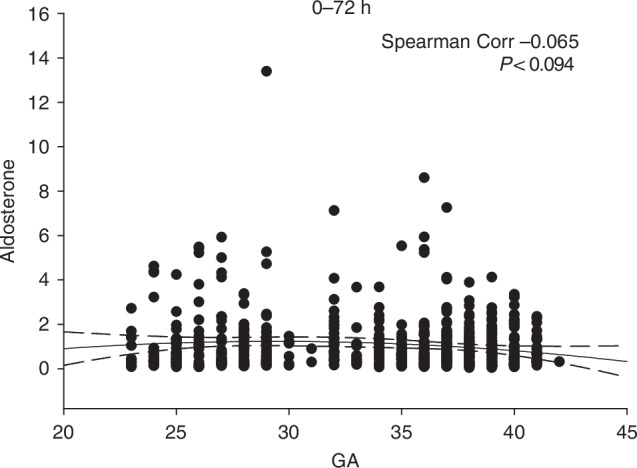
Changes in aldosterone with GA. The DBS data set described is plotted as aldosterone level by GA (in weeks). Data in this figure is confined to the first 72 hours post-delivery. All data in this and subsequent figures is reported in ng/ml. A second order regression line is shown +/− 3 SD, and the Spearman correlation coefficient is as shown.

Aldosterone levels did not correlate significantly with GA, as indicated by a Spearman coefficient near zero (Fig. 3). The spread of aldosterone data across GA groups, including those below 25 and 30 weeks, was comparable to term infants beyond 38 weeks, suggesting minimal developmental variation.

### Cortisol

**Fig. 4:**
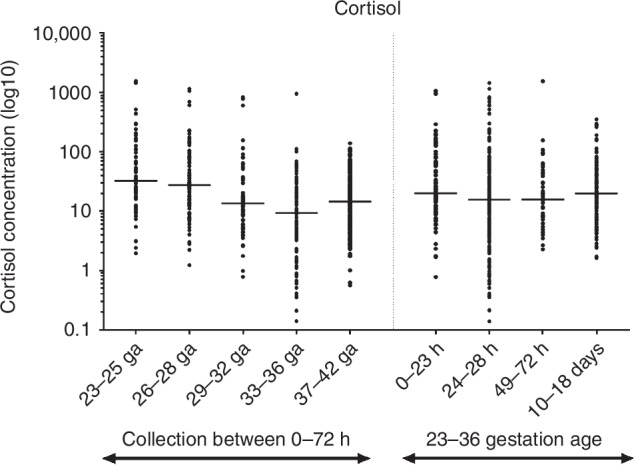
Distribution of cortisol values (ng/ml) in DBS. Individual measured cortisol values in DBS collected from newborns ranging in GA from 23 to 42 weeks, obtained at 0−72 hours of life and 10-18 days of life. The cortisol values are plotted on a log10 scale (y-axis), and the median values for each stratified grouping, based upon GA or collection time, are indicated with the horizontal line.

Cortisol concentrations varied widely among preterm infants (≤36 weeks GA), ranging from 1 to over 1500 ng/mL DBS (~0.2 to 300 μg/dL serum). In term infants, cortisol concentrations were more narrowly distributed, with values between 0.56 and 139 ng/mL DBS. Median cortisol levels in preterm infants demonstrated a modest decline with increasing GA (16.95 ng/mL in preterm vs. 14.45 ng/mL in term infants), but the differences were not statistically significant (*p* = 0.2312). No significant differences were observed by sex, nor were temporal changes seen from birth through 14 days of life (Fig. 4).

**Fig. 5:**
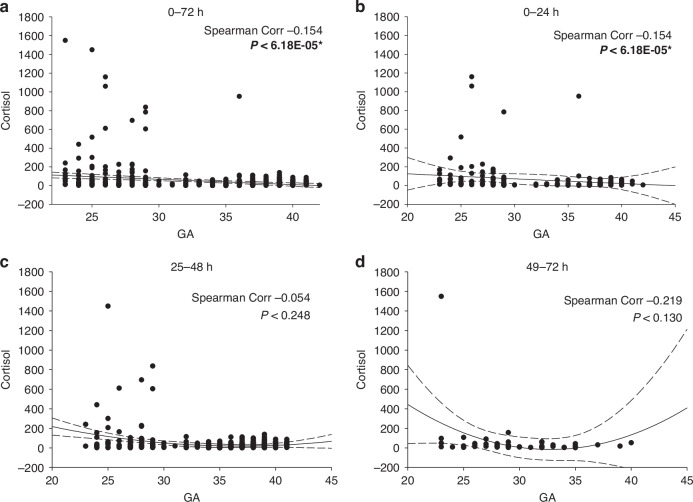
Changes in cortisol with GA. The DBS data set described is plotted as cortisol level by GA (in weeks). Data in panel **a** is the first 72 post-delivery values combined. Panels **b**–**d** are the data in the 0–24, 25–48, and 49–72 hour period post-delivery. All data in this and subsequent figures is reported in ng/ml. A second-order regression line is shown +/− 3 SD, and the Spearman correlation coefficient is as shown in each case. * Indicates significance at *P* < 0.05.

Spearman’s analysis revealed a weak negative correlation (r = −0.154) between cortisol levels and GA during the first 72 hours of life (Fig. 5a). Outlier cortisol values > 200 ng/mL were predominantly observed in infants <30 weeks GA during the 0–24 hour and 25–48 hour windows but were absent by 49–72 hours in all but one very preterm infant (Fig. 5b–d).

### Corticosterone

**Fig. 6:**
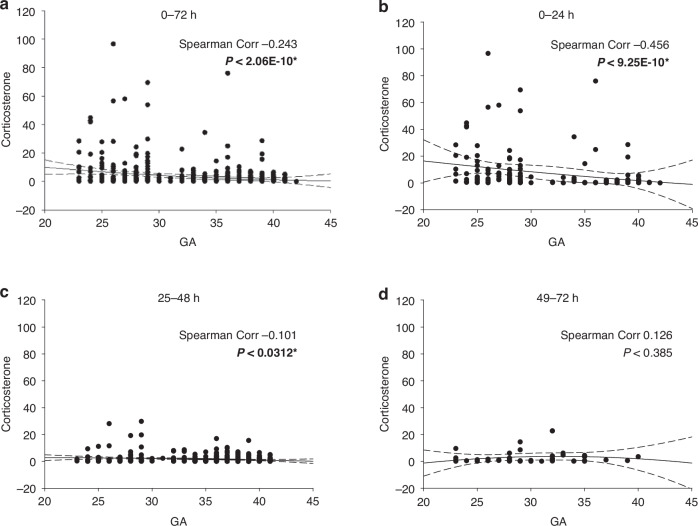
Changes in corticosterone with GA. The DBS data set described is plotted as corticosterone level by GA (in weeks). Data in panel **a** is the first 72 hour post-delivery values combined. Panels **b**–**d** are the data in the 0-24, 25-48, and 49-72 hour period post-delivery. All data in this and subsequent figures is reported in ng/ml. A second-order regression line is shown +/− 3 SD, and the Spearman correlation coefficient is as shown in each case. * Indicates significance at *P* < 0.05.

Moving now to measures of the efficiency of steroid production, it is important to note that in adrenal immaturity or cortisol insufficiency, corticosterone would be the alternate product to cortisol, where p450c17 expression is rate-limiting. Corticosterone levels showed a stronger inverse correlation with GA during the first 72 hours (r = −0.243, Fig. 6), suggesting impaired adrenal efficiency in preterm infants that improved with advancing GA. Among term infants (≥38 weeks GA), corticosterone levels rarely exceeded 40 ng/mL. Conversely, preterm infants <30 weeks GA (*n* = 6) frequently exhibited exaggerated corticosterone levels (>40 ng/mL) compared to infants at 30–38 weeks (*n* = 1). This agrees with the distribution of data for cortisol and suggests despite significant cortisol output in the most extreme cases, immaturity of p450c17 expression exists at 30 weeks GA and below.

### Cortisol to corticosterone ratio

**Fig. 7:**
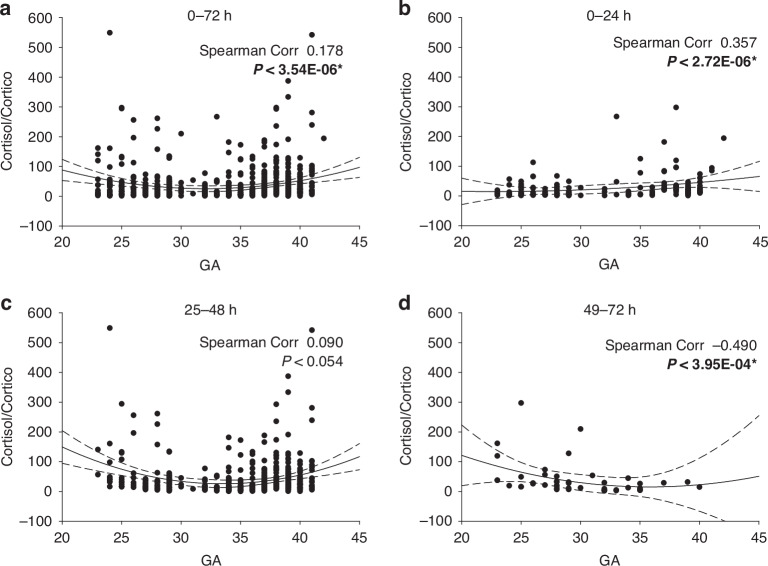
Changes in cortisol/corticosterone ratio with GA. The DBS data set described is plotted as cortisol level/corticosterone level by GA (in weeks). Data in panel **a** is the first 72 hours post-delivery values combined. Panels **b**–**d** are the data in the 0–24, 25–48 and 49–72 hour period post-delivery. All data in this and subsequent figures is reported in ng/ml. A second order regression line is shown +/− 3 SD, and the Spearman correlation coefficient is as shown in each case. * Indicates significance at *P* < 0.05.

The cortisol to corticosterone ratio, a marker of adrenal efficiency, increased with GA, over the initial 72 hours after birth, as shown by a positive Spearman coefficient of 0.178 (Fig. 7a). This suggests a gradual improvement in adrenal efficiency for all subjects during this period. While most data points clustered near the 2nd order correlation line within the 3 SD confidence intervals, several outliers with elevated ratios were observed. During the initial 24-hour period (Fig. 7b), the relationship between the ratio and GA was more pronounced, with a stronger Spearman coefficient of 0.357. Notably, preterm infants (<30 weeks GA) predominantly displayed ratios below the confidence intervals consistent with immaturity of p450c17 expression at birth.

By 25–48 hours (Fig. 7c), ratios began to increase in both preterm (<30 weeks GA) and more mature infants, suggesting induction of p450c17 and improved adrenal efficiency. At this stage, the Spearman’s coefficient was no longer significant, reflecting less variability across GA. By 49-72 hours (Fig. 7d), preterm infants (<30 weeks GA) exhibited high ratios, suggesting they were still adapting to external stressors and gaining control of their environment. In contrast, infants above 30 weeks GA had ratios that remained within confidence limits, indicating more consistent adrenal efficiency.

### 17OHP4

An alternative measure of adrenal *inefficiency* is 17OHP4 (‘17OHP’) which accumulates when cortisol synthesis is insufficient. This occurs due to a strong endocrine drive (elevated ACTH) that stimulates p450c17 and 3ßHSD to convert early substrates into 17OHP4, but limited activity in the ‘late pathway’ enzymes (p450c21 and p450c11) prevents its further conversion to cortisol. In a healthy term infant or adult, these pathways efficiently reduce circulating 17OHP4 to basal levels.

**Fig. 8:**
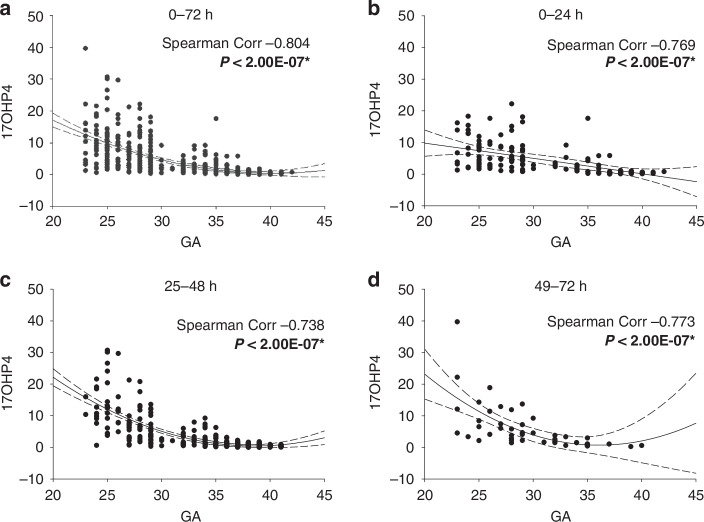
Changes in 17-hydroxyprogesterone (17OHP4) with GA. The DBS data set described is plotted as 17OHP4 level by GA (in weeks). Data in **a** is the first 72 hours post-delivery values combined. Panels **b**–**d** are the data in the 0–24, 25–48 and 49–72 hour period post-delivery. All data in this and subsequent figures is reported in ng/ml. A second order regression line is shown +/− 3 SD, and the Spearman correlation coefficient is as shown in each case. * Indicates significance at *P* < 0.05.

Our analysis over a 72-hour period showed that elevated 17OHP4 levels were most prominent in infants with earlier GA, with a strong negative Spearman’s coefficient of −0.804 (Fig. 8a).

By contrast, levels in infants born at or beyond 38 weeks GA were essentially zero, underscoring 17OHP4 as a sensitive marker of adrenal inefficiency in preterm infants.

When analyzed in 24-hour intervals, the trend remained consistent. During the first 24 hours (Fig. 8b), the Spearman’s coefficient was −0.769, with elevated 17OHP4 levels exceeding the second-order fit and confidence intervals in infants <30 weeks GA. This pattern persisted into the 25–48 hour window (Fig. 8c), with a Spearman coefficient of −0.738 and an even greater spread of data points beyond the confidence intervals for this subgroup. By the 49–72 hour period (Fig. 8d), the coefficient remained strong at −0.773, and outliers continued to be observed exclusively in infants <30 weeks GA. This sustained elevation highlights the significant adrenal inefficiency in the most preterm infants compared to their more mature counterparts.

### Three-dimensional steroid relationships

**Fig. 9:**
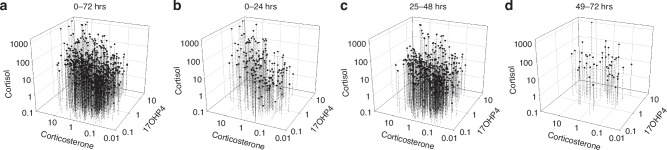
Changes in 17OHP4 vs corticosterone vs cortisol with GA. The DBS data set described is plotted as 17OHP4 vs corticosterone vs cortisol values for all GA subjects. In Panel **a**, we show the data for all samples obtained in the 0–72 hours period after delivery. Panels **b**–**d** are the data in the 0–24, 25–48 and 49–72 hour period post-delivery. All data in this figure is reported in ng/ml.

The observed changes in cortisol levels are primarily dictated by limitations of p450c17 expression, whereas changes in 17OHP4 also reflect the efficiency or inefficiency of the late steroidogenesis pathway. To better understand these relationships, we represented the combined data using a 3D graph (Fig. 9). This representation shows that, regardless of GA, low values of cortisol and corticosterone correspond to low levels of 17OHP4 (data points nearest the observer). In contrast, high levels of both cortisol and corticosterone correlate with high levels of 17OHP4 (data points furthest from the observer). Breaking the data down by 24-hour intervals reveals additional insights. During the first 24 hours post-delivery, the steepness of the 3D surface—from low to high values across these markers—is most pronounced. This steepness diminishes over time, reflecting two key processes: (i) the newborn’s adrenal efficiency as p450c17 and late-pathway metabolic capacity increase, and (ii) all three gene products are induced together by the same cAMP-coupled hormone mediators.^16^

### Cortisol and corticosterone beyond 72 hours

**Fig. 10:**
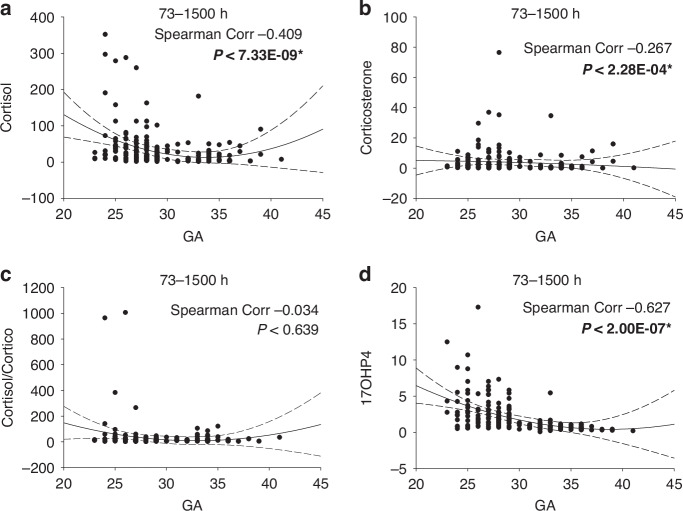
Changes in steroid levels beyond 72 hours with GA. The DBS data set for samples obtained after 72 hours is plotted by GA (in weeks). Data in Panels **a-d** are for cortisol, corticosterone, cortisol/corticosterone ratio, and 17OHP4, respectively. All data in this and subsequent figures is reported in ng/ml. A second-order regression line is shown +/- 3 SD, and the Spearman correlation coefficient is as shown in each case. * Indicates significance at *P* < 0.05.

Analysis of samples collected beyond 72 hours post-delivery reinforced the findings from the first three days (Fig. 10). Extreme cortisol levels (≥1000 ng/mL) observed during the initial 72 hours were no longer present; however, the highest cortisol values, particularly those exceeding 200 ng/mL, continued to be associated with infants born before 30 weeks GA. Corticosterone levels also showed occasional elevations in this group, though less frequently than cortisol. Of note, the cortisol to corticosterone ratio was dramatically increased in a small subset of infants born prior to 30 weeks GA. Additionally, 17OHP4 levels displayed a broad distribution in these early GA infants with values tightening to within 3 SD of the mean as GA increased. The Spearman’s coefficient for cortisol was −0.409, for corticosterone was −0.267, and for 17OHP4 was the highest at −0.627. All were highly significant and fit the concept that those born prior to 30 weeks are ‘distressed’ through adrenal insufficiency, and thereafter, most subjects improved towards near-term values.

## Discussion

Our study delineates the adrenal steroidogenic profiles in preterm infants, identifying distinct developmental shifts in adrenal function. Preterm infants born before 30 weeks GA exhibited elevated cortisol, 17OHP4, and corticosterone levels, which progressively decreased with advancing GA. In contrast, aldosterone levels remained consistent across all GAs. These elevated levels suggest significant stress responses in underdeveloped adrenal glands, emphasizing the need for GA-adjusted reference ranges to improve diagnosis and management of adrenal insufficiency in preterm infants. Our findings provide insights into adrenal insufficiency in preterm infants, with important implications for diagnosis and management in this vulnerable population.

Averaging cortisol levels by GA shows no discernible difference between preterm and term infants. However, Spearman’s analysis reveals a notable trend, corroborated by analyzing early steroid pathway intermediates. Despite the challenge of small sample sizes in extremely preterm cohorts, our study included 65 infants aged 23–25 weeks. Unlike the Premiloc study, which found no correlation between serum cortisol and GA but noted sex differences,^34^ our findings align with research indicating elevated levels of steroidogenic precursors like 17OHP4 and corticosterone in preterm infants.^30,32,35–38^ These results underscore the complex and developing HPA axis in preterm infants. They suggest that, beyond isolated cortisol measurements, tracking additional markers like 17OHP4 and corticosterone is important for assessing adrenal insufficiency and guiding clinical decisions.

Building on our findings, understanding the mechanisms driving these adrenal responses is essential. This requires drawing on literature exploring rate-limiting steps in adrenal steroid biosynthesis under both normal^21^ and pathological conditions.^24,39^ This knowledge can be instrumental in refining the care of extremely preterm infants in neonatal intensive care units.

Our findings challenge the theory that 17OHP4 in utero reflects placental progesterone conversion. The similarity in 17OHP4 levels across the initial 48 hours post-birth, despite placental detachment, suggests an internal adrenal origin rather than an external placental source. We also address the misconception that corticosterone is solely an aldosterone precursor, unrelated to cortisol synthesis. Our data indicate that when adrenal efficiency is compromised (notably due to insufficient p450c17), corticosterone emerges as a significant byproduct of hindered cortisol synthesis. This is directly evidenced by altered cortisol-to-corticosterone ratios, especially with high cortisol production, indicating adrenal stress and potential insufficiency. These observations underscore the complexity of adrenal steroidogenesis in preterm infants. They highlight the need to consider both cortisol production and its interplay with corticosterone and 17OHP4 when assessing adrenal function. In addition, the observed cortisol elevations raise questions about the roles of ACTH, CRH, and potentially unusual adrenergic stimuli in regulating adrenal responses. While direct correlation with these hormones is limited by our data, the findings align with mammalian studies suggesting beta-adrenergic signaling could influence steroidogenesis, particularly under the stress of preterm birth.^40,41^

Our study also clarifies cortisol variability in critically ill newborns, addressing the observed range from “relative” or “transient” adrenal insufficiency to elevated cortisol levels without a clear correlation to clinical signs of adrenal insufficiency.^2–8,42^ Specifically, our findings indicate the HPA axis in extremely preterm infants may be excessively activated by intrauterine, delivery and critical illness stressors. This activation attempts to maintain high cortisol levels via continuous ACTH stimulation of an insufficient adrenal. Furthermore, betamethasone, used to accelerate surfactant production, may impair adrenal maturation by negatively blocking ACTH, which normally induces p450c17 and p450c11 expression.

The effectiveness of high cortisol levels for survival remains uncertain; some evidence links elevated cortisol to increased morbidity and mortality,^15,43^ particularly with postnatal steroid treatment.^44^ The precise impact of factors like stress, hypoxia, and intracranial bleeding on the HPA axis and the subsequent cortisol response remains to be fully understood, potentially affecting the interplay between central (ACTH/CRH-related) and secondary (adrenal immaturity-related) adrenal insufficiency in preterm infants. The possibility that an adrenergic stimulus beyond ACTH/ CRH influences cortisol synthesis, particularly if given exogenously after birth, merits further exploration. A response mechanism involving catecholamines like epinephrine and norepinephrine is plausible with severe prematurity, especially considering the incomplete adrenal cortex and medulla zonation in infants under 30 weeks GA.

While low cortisol has traditionally signaled illness in infants, recent findings show that elevated cortisol levels—up to ten times higher in preterm compared to term infants—may also pose risks, potentially disrupting feedback mechanisms and causing electrolyte imbalances. Our results prompt the question: is this heightened cortisol response protective or harmful in preterm infants? These elevated levels suggest a possible loss of feedback control or an alternative cortisol-driving mechanism needing medical intervention to mitigate glucocorticoid and mineralocorticoid receptor-related side effects.

Despite high cortisol levels, glucocorticoid receptor (GR) -mediated activities are likely maintained. However, the mineralocorticoid receptor (MR), which binds both cortisol and aldosterone, is a concern.^45^ Supraphysiologic cortisol levels risk overwhelming the MR, potentially leading to receptor instability and electrolyte imbalance, a critical factor in preterm hyperkalemia. Treatment strategies may need to address the pituitary feedback loop or block adrenergic stimulation to normalize excess cortisol production, especially given the association between high cortisol levels and increased adverse outcomes in preterm infants treated with hydrocortisone.^34,44^ Additionally, the influence of beta-adrenergic triggers and the effects of various medications on the underdeveloped adrenal physiology of extremely preterm infants warrant further investigation. Synthetic glucocorticoids that do not engage the MR might mitigate these risks, and beta-blockers may help regulate persistent cortisol overproduction. Such approaches aim to maintain glucocorticoids’ lung-protective effects while safeguarding against the negative impacts of excessive cortisol on MR function.

Initiated by clinical observations of adrenal insufficiency in extremely preterm infants, our study also explored aldosterone levels, which, unlike cortisol, are not regulated by pituitary ACTH. Our findings reveal that prematurity minimally impacts fetal aldosterone production capacity. This aligns with prior research indicating minor aldosterone variations across GAs.^14,46,47^ This likely occurs because aldosterone synthesis does not rely on p450c17, contrasting with cortisol’s large production volume. Our results support that p450aldo is the primary rate-limiting enzyme in the mineralocorticoid pathway, and that aldosterone does not influence ACTH production feedback.^21^ Notably, a slight aldosterone reduction was observed in infants between 23–25 weeks and during the first 24 hours post-birth, potentially linked to betamethasone effects if administered within 72 hours before delivery.^48^ Further research on MR expression and functionality in very preterm infants is essential to enhance our understanding of their unique endocrine landscape.

Our study has several limitations with clinical implications. Although the data set for infants born at 23-25 weeks’ GA is small, observed trends in this subgroup align with older GA groups. One limitation is the inability to correlate steroid levels with clinical indicators (e.g., critical illness, adrenal insufficiency, acute kidney injury with reduced renal cortisol clearance, or prenatal factors such as growth restriction or infection). Previous studies linked higher serum cortisol levels in the first 24 hours after birth and chorioamnionitis.^34^ Another consideration is the potential impact of antenatal or postnatal steroid treatment on the developing HPA axis and cortisol levels. Betamethasone, given to >80% of mothers prior to delivery for lung maturation, crosses the placenta and enters fetal circulation.^49^ While LC-MS/MS distinguishes betamethasone from endogenous cortisol, antenatal betamethasone could transiently alter newborn steroid profiles depending on dose and timing.^34,50–52^ Preterm babies delivered within 72 hours of maternal betamethasone are predicted to have transiently lower initial cortisol and aldosterone levels, with the impact diminishing beyond this window.^11,12,48^ Finally, this study shows GA-adjusted steroid profiles at two time points but could not track trends in individual preterm infants.

Another important limitation is the inability to account for postnatal hydrocortisone or fludrocortisone use, as our deidentified dataset precludes linking steroid profiles to clinical treatments. LC-MS/MS cannot distinguish hydrocortisone from endogenous cortisol production due to identical mass to charge ratios and retention times. Our clinical experience and reports like the Premiloc study suggest that elevated serum cortisol occurs in extremely preterm babies prior to receiving hydrocortisone, even with signs of adrenal insufficiency.^34,44^ While exogenous steroids could influence cortisol and related precursor levels, the observed corticosterone and 17OHP4 elevations, consistent with adrenal immaturity, suggest that these findings are more likely attributable to endogenous adrenal dysfunction. Future studies integrating detailed clinical data, including antenatal stressors and postnatal steroid administration, will help distinguish exogenous treatments effects from intrinsic adrenal physiology in preterm infants.

Our study highlights the clinical significance of elevated steroidogenic precursors (17OHP4 and corticosterone) in preterm infants, indicating an active but potentially inefficient adrenal gland attempting cortisol production. These elevations, traditionally associated with adrenal immaturity or congenital enzyme deficiencies (diagnosed using 17OHP4), might also indicate adrenal insufficiency in preterm infants. The cortisol/corticosterone ratio and elevation of 17OHP4, particularly when assessed within 24 hours post-delivery, serves as a key indicator of the newborn HPA axis’s inability to fully respond to stress, and offer insights into the specific adrenal enzyme efficiency. This study introduces the novel perspective that the preterm HPA axis can respond dynamically to birth and subsequent stress longer term. As LC-MS/MS availability expands in neonatology and pediatrics, the long-term implications of these findings on adrenal development and function warrant further exploration.

Advances in neonatology have historically outpaced our understanding of the complex and changing physiology of the extremely preterm infant, partly due to blood sampling difficulties. Our research demonstrates that LC-MS/MS analysis of steroid profiles from minimal blood volumes enhances our understanding of the HPA axis alongside other developing organs such as the lung and brain. This approach could refine hydrocortisone therapy in preterm infants, leveraging LC-MS/MS techniques already applied in newborn screenings.^24,30,32,33,53–55^ Recent studies suggest LC-MS/MS accurately measures adrenal steroid reference ranges in term infant venous blood, showing profiles differ from preterm infants.^56^ Reference levels of 17OHP4 and corticosterone in healthy term newborns were higher at 7-14 days compared to 3-7 days.^56^ Our study and others, when viewed from a perspective of adrenal development, explain variable cortisol levels across GA and illness, underscoring the value of understanding cortisol levels in the context of its biosynthetic precursors, particularly in extremely preterm infants.

Future studies should establish normative cortisol/corticosterone ratios and 17OHP4 levels, accounting for variables like GA, body weight, post-delivery timing, and clinical illness. Documenting preterm infants’ response to an ACTH challenge will help differentiate central adrenal insufficiency due to inadequate ACTH/CRH from secondary insufficiency due to adrenal immaturity. Although steroid reference ranges do not decrease the importance of clinical signs of adrenal insufficiency in making treatment decisions,^57^ they may offer early insight into adrenal deficits, allowing modification of postnatal steroid timing, dose, and duration. Our next steps are to compare steroid profiles from capillary or venous samples in extremely preterm infants, including data on prenatal risk factors such as asphyxia and chorioamnionitis and clinical signs of adrenal insufficiency.

### Data sharing

This study utilized de-identified residual DBS specimens from newborn screening programs. Due to the rarity of conditions studied and the potential for re-identification, sharing the dataset could compromise patient privacy. Therefore, the data will not be publicly available. Researchers with specific inquiries may contact the corresponding author, subject to institutional and ethical approvals.

## Supplementary information

Supplemental Table 1

Supplemental Table 2
