# Supplementary material for: Elevation of corticosterone and 17OH progesterone in extremely preterm infants and clinical implications
Source: Pediatr Res. 2025 Aug 7;99(3):1085–95. doi: 10.1038/s41390-025-04216-5 (PMC13021513; doi:10.1038/s41390-025-04216-5)
Supplement: Supplementary file 1 — Supplemental Table 1 [file 41390_2025_4216_MOESM1_ESM.pdf]

Supplemental Table 1: Parameters for detection of steroids and internal standards

Supplemental table 1 provides the MRM transitions, retention times, collision energy, declustering potential, excitation potential, and collision cell exit potential (CXL) for each steroid and its corresponding internal standard. MRM=multiple reaction monitoring.

Steroids and Internal Standards	MRM Transitions	Retention Time (min)	Collision Energy (V)	Declustering Potential	Excitation Potential	CXL
Aldosterone	361.2→343.1	5.7	24	85	10	17
D8-Aldosterone	369.2→351.2	5.7	24	110	10	13
Androstenedione	287.2→97.0	7.2	28	70	10	13
D7-Androstenedione	294.2→100.1	7.2	27	70	10	13
Corticosterone	347.2 →121.0	6.4	21	50	10	13
D4-Corticosterone	351.2→333.1	6.4	22	100	10	12
Cortisol	363.3→121.2	5.2	30	70	10	13
D4-Cortisol	367.1→121.1	5.2	34	70	10	13
11-Deoxycortisol	347.3→97.0	6.2	51	70	10	13
D2 11-Deoxycortisol	349.1→109.0	6.2	45	70	10	13
17-hydroxyprogesterone	331.2→97.0	6.7	60	70	10	13
D8-17-hydroxygrogesterone	339.2→113.1	6.7	47	70	10	13
Progesterone	315.2→97.1	8.0	24	90	10	16
D9-Progesterone	324.2→100.1	8.0	25	100	10	13
