# Supplementary material for: Elevation of corticosterone and 17OH progesterone in extremely preterm infants and clinical implications
Source: Pediatr Res. 2025 Aug 7;99(3):1085–95. doi: 10.1038/s41390-025-04216-5 (PMC13021513; doi:10.1038/s41390-025-04216-5)
Supplement: Supplementary file 2 — Supplemental Table 2 [file 41390_2025_4216_MOESM2_ESM.pdf]

**Supplemental Table 2: Liquid chromatography profile**

Supplemental table 2 provides the liquid chromatography injection profile for separation of the steroids. %B=percentage of mobile phase B at the corresponding time.

| Time (min) | %B |
|---|---|
| 0.01 | 35 |
| 0.50 | 35 |
| 5.00 | 85 |
| 9.50 | 100 |
| 13.00 | 100 |
| 13.50 | 35 |
